# Who Still Gets Ligated? Reasons for Persistence of Surgical Ligation of the Patent Ductus Arteriosus Following Availability of Transcatheter Device Occlusion for Premature Neonates

**DOI:** 10.3390/jcdd11050132

**Published:** 2024-04-23

**Authors:** Julia K. Hoffmann, Zahra Khazal, Wievineke Apers, Puneet Sharma, Constance G. Weismann, Kira Kaganov, Craig R. Wheeler, Michael Farias, Diego Porras, Philip Levy, Sarah U. Morton

**Affiliations:** 1 Division of Newborn Medicine, Department of Pediatrics, Boston Children’s Hospital, Boston, MA 02115, USAphilip.levy@childrens.harvard.edu (P.L.); 2 Department of Pediatric Cardiology, Skåne University Hospital, Department of Clinical Sciences, Lund University, 221 00 Lund, Sweden; 3 Department of Pediatric Cardiology and Pediatric Intensive Care, Ludwig Maximilian University, 80539 Munich, Germany; 4 Department of Neonatology, Dana Dwek Children’s Hospital, Tel Aviv Sourasky Medical Center, Tel Aviv 6423906, Israel; 5 Departments of Respiratory Care and Extracorporeal Membrane Oxygenation, Boston Children’s Hospital, Boston, MA 02115, USA; 6 Department of Cardiology, Boston Children’s Hospital, Boston, MA 02115, USA

**Keywords:** patent ductus arteriosus, PDA, surgical ligation, prematurity

## Abstract

(1) Background: To identify reasons for the persistence of surgical ligation of the patent ductus arteriosus (PDA) in premature infants after the 2019 Food and Drug Administration (FDA) approval of transcatheter device closure; (2) Methods: We performed a 10-year (2014–2023) single-institution retrospective study of premature infants (<37 weeks) and compared clinical characteristics and neonatal morbidities between neonates that underwent surgical ligation before (epoch 1) and after (epoch 2) FDA approval of transcatheter closure; (3) Results: We identified 120 premature infants that underwent surgical ligation (*n* = 94 before, *n* = 26 after FDA approval). Unfavorable PDA morphology, active infection, and recent abdominal pathology were the most common reasons for surgical ligation over device occlusion in epoch 2. There were no differences in demographics, age at closure, or outcomes between infants who received surgical ligation in the two epochs; (4) Conclusions: Despite increasing trends for transcatheter PDA closure in premature infants, surgical ligation persists due to unfavorable ductal morphology, active infection, or abdominal pathology.

## 1. Introduction

Surgical ligation rates of the patent ductus arteriosus (PDA) have declined over the past two decades in premature infants [1,2,3,4,5,6,7]. Contemporary evidence suggests that the temporal shifts away from surgical ligation rates resulted from the appreciation that spontaneous closure of the ductus occurred more frequently than once expected [8,9] and negative associations with adverse neonatal and neurodevelopmental outcomes [9,10,11,12]. The trend has been more pronounced since the 2019 United States Food and Drug Administration (FDA) approval of a transcatheter device to occlude the PDA in premature infants with the rapid increase in institutions capable of performing device closures [1,2,3]. However, despite the recognition that catheter-based interventions are safer with less morbidity and mortality compared to surgical ligation [13,14,15,16], some premature infants still undergo surgical ligation as the primary choice for definitive closure [1,2,3].

A premature infant that requires definitive procedural closure of a PDA is not eligible for device occlusion if they have an active infection, an intracardiac thrombus, evidence of coarctation of the aorta, or the ductus is too wide or too short [17]. Despite these known contraindications, there has been little focus on understanding why some premature infants still undergo surgical ligation. Accordingly, the primary objective of this study was to explore the reasons for the persistence of surgical ligation following the widespread availability of transcatheter occlusion devices for premature infants and compare the characteristics of infants who received surgical ligation before and after FDA approval of device closures in 2019. The secondary objective was to compare characteristics between infants who underwent surgical ligation and device closure after 2019. This information will be vital for preparing clinicians when discussing indications for definitive procedural approaches to close the PDA in premature infants.

## 2. Materials and Methods

We performed a 10-year (2014–2023) single-institution retrospective case–control study between two-time epochs of all premature infants delivered <37 weeks gestational age who underwent surgical ligation of the PDA at Boston Children’s Hospital. Our center is a high-volume level IV neonatal intensive care unit (NICU) surgical center that serves as a referral institution from 16 hospitals in New England for definitive closure of the PDA. For this study, we excluded premature infants with complex congenital heart disease (except hemodynamically insignificant ventricular septal defects or atrial septal defects) and known genetic or congenital anomalies. Infants with concern or confirmed cortication of the aorta of left pulmonary artery stenosis were not included in this study. The institutional review board of Boston Children’s Hospital approved the study.

Prior to the FDA’s approval of a transcatheter device (Amplatzer PiccoloTM Occluder, Abbott Laboratories, Chicago, IL, USA) to occlude the PDA 2019, surgical ligation was the primary method for definitive closure for premature infants at our institution. In our center, surgical ligation is performed in the operating room and not at the bedside. Patients are intubated in the NICU and transported to the operating room prior [17]. They receive routine anesthesia and muscle relaxation. Intubation is most commonly performed in the NICU, and all patients on high-frequency ventilation must demonstrate stable gas exchange on conventional ventilation prior to transportation for the procedure. After 2019, transcatheter device closure was offered to all premature infants older than 3 days without active infection (defined as a positive blood culture on antibiotics), intracardiac thrombus, clinical or echocardiographic evidence of cardiac output dependence on right to left shunt through the PDA, echocardiographic evidence of coarctation of the aorta or left pulmonary artery stenosis, and a ductus that was <3 mm in length and <4 mm in diameter [16]. In our institution, we offer the Amplatzer Ductal Occluder (Abbott, Santa Clara, CA, USA), Medtronic Microvascular Plug or the Amplatzer Vascular Plug for device occlusion in premature infants with a weight <2.5 kg [18]. The choice of the specific device is at the discretion of the interventional cardiologist. All infants are intubated in the NICU and transported to the cardiac catheterization suite per standard of care [18]. Device placement is not performed at the bedside in our institution. We do not have a lower weight limit for the placement of the device. If patients did not meet these criteria, they were referred for surgical ligation. We documented the reasons why a premature infant did not meet eligibility for device closure and underwent surgical ligation.

In September 2019, we established a multidisciplinary referral program for PDA closure in premature infants at Boston Children’s Hospital [15]. Additionally, we created a de-identified registry that includes all premature infants referred for evaluation for definitive closure of their PDA at our institution from 2014 onward. All data were collected using a structured form and entered into a clinical registry database created in REDCap (Vanderbilt University, Nashville, TN, USA). Demographic data and clinical factors prior to the PDA procedure (e.g., gestational age at referral, prior pharmacological course to attenuate the PDA, age at definitive closure, and common neonatal morbidities) were collected from the medical record of each premature infant. A detailed collection of perinatal characteristics was performed to compare common morbidities of prematurity that arose or were acquired prior to enrollment. Ventilator parameters and physiologic variables were also extracted from the medical record pre-procedure and post-procedure.

We compared demographics, clinical characteristics, and common neonatal morbidities between infants who underwent surgical ligation before (epoch 1: 2014–2019) and after transcatheter closure became available for premature infants at our center in September 2019 (epoch 2: 2019–2023). We classified the morphology of the PDA based on the Krichenko classification [19]. Moreover, we documented the number of transcatheter closures performed in epoch 2 and compared demographics and clinical characteristics between premature infants who underwent device occlusion and surgical ligation.

Descriptive statistics were calculated for both the epochs. Continuous variables were expressed as mean ± standard deviation (SD) or median (interquartile range [IQR] and range) as appropriate. Categorical variables were summarized using percent (count) and compared between epochs using Fisher’s exact test and Pearson’s chi-square test as appropriate. Continuous variables were assessed using the Wilcoxon rank sum test. Data analysis was performed using the R Statistical Package (University of Auckland, Auckland, New Zeeland). All tests were two-sided and *p* values < 0.05 were considered significant. 

## 3. Results

### 3.1. Demographics

**Figure 1:**
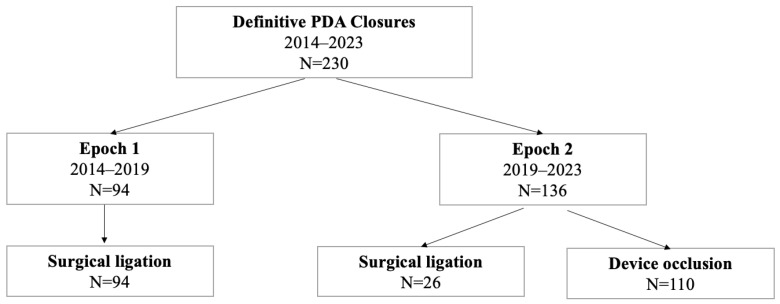
Study cohort.

**Figure 2:**
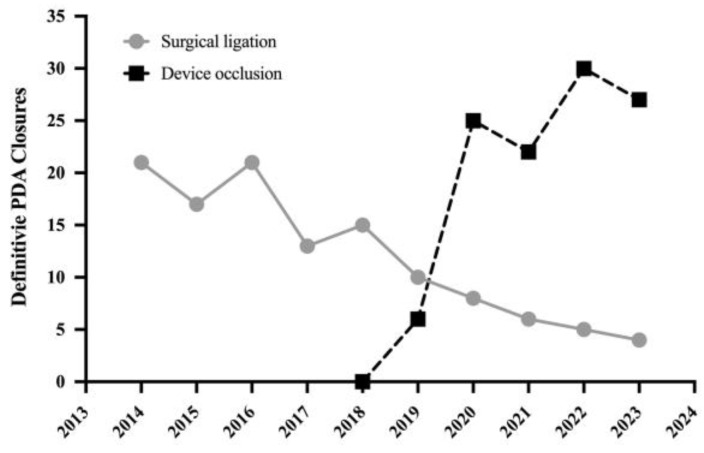
The trend in patent ductus arteriosus (PDA) closure volume over time.

We identified 230 premature infants that had definitive closure of the PDA between 2014 and 2023 in our institution (Figure 1). In epoch 1, from 2014–2019, 94 infants (18.8 infants per year) underwent surgical ligation. In epoch 2, from 2019–2023, 26 infants (5.2 infants per year) underwent surgical ligation at Boston Children’s Hospital. In epoch 2, 110 transcatheter procedures were performed in premature infants with rates of device closure surpassing surgical ligation in our center by 2020 (25 device closures vs. 8 surgical ligations in 2020) (Figure 2).

### 3.2. Epoch Comparisons

There were no differences in median (interquartile range) gestational age at delivery [25 (24, 26) vs. 24 (24, 26) weeks, *p* = 0.224], birthweight [750 (633, 900) vs. 685 (625, 793) grams, *p* = 0.339], necrotizing enterocolitis (35% vs. 24%, *p* = 0.293), or intraventricular hemorrhage (49% vs. 44%, *p* = 0.661; Table 1) when comparing both epochs of surgical ligation. The number of courses of pharmacotherapy prior to definitive closure was similar between epochs [2 (1, 2) vs. 1 (1, 2), *p* = 0.671]. There was a trend towards older age at closure with surgical ligation in epoch 2 [21 (15, 29) days vs. 28 (20, 41) days, *p* = 0.055]. There were no differences in the incidence of high-frequency ventilation (43% vs 35%, *p* = 0.5) or the duration of mechanical ventilation [14 (7,24) vs. 17 (6,41) days, *p* = 0.3], (Table 1).

### 3.3. Reasons for Surgical Ligation in Epoch 2

The reasons for surgical ligation over transcatheter device occlusion in epoch 2 are listed in Table 2. Nine infants (35%) had an active infection, defined as culture-positive bacteremia, and were still on antibiotics. Eight infants (31%) had unfavorable PDA morphology, defined as type B window-like Krichenko PDA with a length of <3 mm (insufficient length to accommodate the entire device inside the PDA), a diameter >4 mm, or concern for hypoplastic aortic arch. Seven (31%) infants had recent abdominal pathology, defined as Bell’s Stage II necrotizing enterocolitis within two weeks of referral or spontaneous intestinal perforation with a Penrose drain. Of the 26 patients, two were eligible for device closure but instead received a surgical ligation. One infant had a pre-procedural complication (tracheal tear prior to the start of the case) and the other infant had intra-procedural technical challenges (inability to place the device during the procedure). There were three infants that were referred to our center in epoch 2 and determined to be eligible for device closure, but their respiratory status prevented safe transfer. These infants underwent surgical ligations at the referral centers and were not included in the final analysis. In epoch 2, we performed a sub-analysis by reason for choosing surgical ligation over device closure. We found that infants who received surgical ligation for abdominal pathology were younger and smaller at the postnatal age of closure compared to those who had morphological contraindications or bacteremia (Supplementary Table S1).

### 3.4. Comparisons between Surgical Ligation and Device Closure in Epoch 2

Compared to infants that underwent device occlusion in epoch 2, infants with surgical ligation had lower median (interquartile range) gestational age at delivery [25 (24, 27) vs. 24 (24, 26) weeks, *p* = 0.042] and smaller birthweight [815 (670, 1010) vs. 685 (625, 793) grams, *p* = 0.016]. (Table 3). The number of courses of pharmacotherapy was the same between the surgical ligation and device closure groups [2 (1, 3) vs. 1 (1, 2), *p* = 0.054]. The postnatal age at closure was lower in the surgical ligation group [41 (26, 68) days vs. 28 (20, 41) days, *p* = 0.013]. When restricted to infants with procedure weight less than 1200 grams, the weight at procedure remained lower in the ligation group birthweight [890 (723, 1000) vs. 1000 (900, 1100) grams, *p* = 0.008] but there were no longer any differences in the frequency of high frequency ventilation (Table 4). 

## 4. Discussion

Transcatheter device occlusion of a PDA can now be safely performed in premature infants [14,17,20]. Despite the national trends away from surgical ligation [1,2,3], it remains a viable alternative for definitive closure if a transcatheter device closure cannot be performed. In this study, we focused on understanding why premature infants underwent surgical ligation of the PDA in this new era of device occlusion availability [17]. We identified that unfavorable ductal morphology, active infection, and recent abdominal pathology were the most common indications for surgical ligation at our center. Additionally, a subset of infants was unstable for transport to our center and underwent surgical ligation at their delivery center. We found no differences in the characteristics of infants who received surgical ligation pre- and post-availability of device occlusion. Infants who underwent surgical ligation after 2019 were of smaller birth weight, younger gestational age, and a lower chronologic age compared to infants who had device occlusion of the PDA. As more literature focuses on comparing outcomes between infants who underwent ligation vs. device [13,16,21], it is imperative that we also focus on understanding why infants still undergo ligation. This knowledge is crucial for providers and families when considering all options for the closure of a hemodynamically significant PDA (hsPDA) in premature infants.

In our institution, device occlusion replaced surgical ligation as the primary method of definitive closure after FDA approval in 2019, and by 2020 more infants underwent transcatheter closure [22]. This clinical practice change reflects current trends from other centers [23,24] and larger population cohort reports [1,2,3]. It is not surprising that the reason for continued surgical ligation in our center mirrors the contraindications for device closure, namely active infection and inadequate PDA morphology. Although advances in infection prevention have been made in the adult literature with the placement of cardiac devices (e.g., pacemakers, valve repairs) [25], we employed a conservative approach by excluding patients from device closure who were receiving antibiotics for bacteremia or medical/surgical necrotizing enterocolitis. Of note, we did place one device in an infant who subsequently tested positive for fungemia within 48 h of device placement. The infant received six weeks of intravenous anti-fungal treatment with no observed sequela (e.g., no intra- or extra-cardiac vegetations or abscesses). While we could not definitely state that there was no association between device placement and this result, this was the only case of a bloodstream infection post-procedure within 48 h of placement.

Over the last decade, more premature infants have gained access to transcatheter device closure either at a regional referral center [1,2,3], with improved transfer processes to a referral center [18], or with a PDA closure team traveling to the birth center [26]. At some large referral centers, PDA closure teams can perform the device occlusions at the bedside and negate the need to travel from the neonatal intensive care unit (NICU) to the catheterization suite [27]. However, there remains a barrier to device closure for those infants who require transfer to a regional center. In our study, three infants were referred to our regional center, and their PDA was deemed amenable to device closure. However, their respiratory requirements prevented safe transport and a surgical ligation was performed at the delivery institution. Some centers have begun to perform the device occlusions at the bedside and therefore negate the need to travel from the birth NICU to the catheterization suite at a referral center [26]. In our center, we do not yet perform the procedure at the bedside nor does our PDA closure team travel to regional NICUs to perform the procedure. Ultimately, to expand access to this beneficial procedure, we feel both options are areas of future growth. Additionally, the use of high-frequency ventilation strategies was once thought to be a barrier to performing any definitive closure techniques, but recent evidence has shown that device closure of the PDA in an infant supported by high-frequency jet ventilation is feasible and safe [28]. This could potentially increase the number of patients eligible for device closure, however, it is worth noting that no infants were excluded from device closure in our study due to high-frequency ventilation.

In this study, premature infants who received surgical ligation after 2019 instead of device closure had smaller birthweights and were at a younger postnatal age at the time of definitive closure, which was even more pronounced in infants with abdominal pathology. In our institution, a lower weight < 700 g was not a contraindication for device occlusion consideration [14], and a recent study from the Improving Pediatric and Adult Congenital Treatments (IMPACT) registry showed 100% technical success with placement of devices below this weight. In our study, infants who underwent surgical ligation compared to device closure trended to receive fewer pharmacotherapy courses prior to definitive closure. We suspect that the reasons premature infants currently receive a device to occlude their PDA at older postnatal ages (and larger weights) are due to the recent push towards initial supportive non-pharmacological approaches [8,29], expected learning curve with building a transcatheter device closure program [24], or general lack of familiarity with device closure options. Our results are consistent with those of Lai et al. [1] and Shah et al. [2] who both documented an increase in age at PDA diagnosis and older age at definitive closure with device occlusion compared with surgical ligation. As more centers become aware of device closure and develop a standardized approach for consideration, the postnatal age at referral and postnatal age at closure may drop over time and this will directly impact the medical approaches to attenuate a hsPDA. This is of the utmost importance as previous literature has demonstrated improved outcomes (less respiratory support, lower incidence of pulmonary hypertension, and improved weight gain) when the device closure procedure is performed under 4 weeks of age [30,31].

The current state of the field is centered on understanding overall trends of definitive procedural closure [1,2,3], comparing short- and long-term outcomes between surgical ligation a device occlusion [12,32], and exploring a prospective outcomes-based randomized controlled trial (PIVOTAL NCT05547165) to determine the effectiveness of treatments for the “high-risk” premature infants with a hsPDA. In comparison to device closure, infants undergoing surgical ligation have more respiratory instability with a greater need for high-frequency ventilation after the procedure [15,21], take longer to wean to extubation, have a higher incidence of post-closure cardiac compromise (aka low cardiac output syndrome) [15,19,33,34], have longer intensive care and hospital lengths of stay, higher costs [13], and increased mortality [13,21]. The awareness of these complications makes it even more relevant to identify the premature infant that still undergoes surgical ligation and use our results to revisit the relative and absolute contraindications for device closure that is guided by outcomes-based evidence.

The implications of this study should be interpreted within the framework of the study’s limitations. Our relative indications for surgical ligation, namely active infection and recent abdominal pathology are based on local experience and would benefit from randomized clinical trials to assess the appropriateness of these guidelines. In our center, all definitive closures occur in the operating room or the catheterization suite, but we recognize that several other institutions offer bedside procedures, and this could play a role in understanding who might still receive a surgical ligation over device occlusion. The patient-specific demographic and clinical data are restricted to what was available in the registry. We did not collect any information regarding parental concerns about radiation exposure, and there were no incidences of acute kidney injury reported in our study. In addition, causality cannot be proven from this descriptive retrospective study of observed trends. We did utilize a standardized qualitative echocardiography protocol to detect a PDA prior to definitive closure, but there is no agreed definition of a PDA that warrants treatment. 

## 5. Conclusions

In our institution, transcatheter device intervention is now offered as first-line intervention for definitive closure of a PDA in premature infants, which were traditionally treated with surgical ligation. Premature infants may still undergo surgical ligation if there is concern over ductal morphology, active infection, or ongoing abdominal processes. This information can inform decision-making and facilitate planning for definitive closure, as well as generate interest in the advancement of device design and/or procedural approaches aimed at increasing eligibility for transcatheter closure of the PDA.

## Figures and Tables

**Table 1:** Surgical ligation epoch comparisons.

Characteristics	Epoch 1 2014–2019 N = 94	Epoch 2 2019 ^1^–2023 N = 26	*p*-Value ^2^
Demographics			
Gestational age (weeks)	25 (24, 26)	24 (24, 26)	0.224
Birth weight (grams)	750 (633, 900)	685 (625, 793)	0.339
Birth weight Z-scores	0.24 (−0.48, 0.80)	0.13 (−0.31, 0.69)	0.949
Sex (female)	37 (39%)	14 (54%)	0.186
Age at procedure (days)	21 (15, 29)	28 (20, 41)	0.055
Postmenstrual age at procedure (weeks)	29 (27, 31)	28 (26, 30)	0.100
Weight at procedure (grams)	900 (740, 1180)	950 (760, 1185)	0.652
Procedure weight Z-scores	−1.01 (−1.44, −0.62)	−1.13 (−1.48, −0.85)	0.317
Pharmacotherapy before closure	79 (84%)	20 (77%)	0.394
#of pharmacotherapy courses	2 (1, 2)	1 (1, 2)	0.671
Common comorbidities prior to closure			
Necrotizing enterocolitis (≥Bells Stage II)	33 (35%)	6 (24%)	0.293
Intraventricular hemorrhage (any IVH) Grade III/IV IVH	46 (49%) 22 (23%)	11 (44%) 6 (24%)	0.661 0.950
Post-procedural respiratory outcomes			
High frequency ventilation	41 (44%)	9 (38%)	0.588
Duration of mechanical ventilation (days)	13 (7, 24)	17 (6, 38)	0.248

Data is reported as. Median (IQR) or n (%). ^1^ We launched our Premature PDA Closure program on 1 September 2019, after the FDA approval for device occlusion in extreme premature infants in January 2019. ^2^ Wilcoxon rank sum test; Pearson’s Chi-squared test; Fisher’s exact test.

**Table 2:** Reasons for surgical ligation in epoch 2.

Reasons	Number (Percentage)
Active infection ^1^	9 (35%)
Unfavorable morphology ^2^	8 (31%)
Recent abdominal pathology ^3^	7 (27%)
Other ^4^	2 (7%)

Data is reported as n (%). ^1^ Defined as culture-positive bacteremia and on antibiotics at the time of definitive closure consideration. ^2^ Defined as echocardiographic evidence of cortication of the aorta or left pulmonary artery stenosis, and a ductus that was <3 mm in length and <4 mm in diameter. ^3^ Defined as Bells Stage II necrotizing enterocolitis within two weeks of referral or spontaneous intestinal perforation with a Penrose drain. ^4^ One infant had a pre-procedural complication prior to the case. A second infant had unsuccessful placement of a device.

**Table 3:** Comparison between infants who received device occlusion vs surgical ligation after 2019.

Characteristics	Device ^1^ (N = 110)	Ligation ^1^ (N = 26)	*p*-Value ^2^
Demographics			
Gestational age (weeks)	25.00 (24, 27)	24 (24, 26)	0.042
Birth weight (grams)	815 (665, 1010)	685 (625, 793)	0.016
Birth weight Z-scores	0.34 (−0.35, 1.26)	0.13 (−0.31, 0.69)	0.419
Sex (female)	60 (56%)	14 (54%)	0.837
Age at procedure (days)	41 (26, 68)	28 (20, 41)	0.013
Postmenstrual age at procedure (weeks)	31 (28, 36)	28 (26, 30)	0.002
Weight at procedure (grams)	1380 (1100, 2330)	950 (760, 1185)	<0.001
Procedure weight Z-scores	−0.77 (−1.11, −0.10)	−1.13 (−1.48, −0.85)	0.007
Pharmacotherapy before closure	96 (87%)	20 (77%)	0.217
#of pharmacotherapy courses	2.00 (1.00, 3.00)	1.00 (1.00, 2.00)	0.054
Common comorbidities prior to closure			
Necrotizing enterocolitis (≥Bells Stage II) ^3^	14 (13%)	6 (24%)	0.211
Intraventricular hemorrhage (any IVH) Grade III/IV IVH	42 (39%) 15 (14%)	11 (44%) 6 (24%)	0.663 0.223
Baseline respiratory/hemodynamic status			
Use of inotropic	5 (9.8%)	2 (14%)	0.638
Oxygen saturation index	4.6 (2.7, 7.5)	4.1 (2.0, 6.5)	0.705
Post-procedural respiratory outcomes			
High frequency ventilation	11 (12%)	9 (38%)	0.013
Duration of mechanical ventilation (days)	26 (8, 51)	17 (6, 38)	0.424

Data is reported as. Median (IQR) or n (%). ^1^ We launched a PDA Closure program on 1 September 2019, after the FDA approval for device occlusion in extreme premature infants in January 2019. ^2^ Wilcoxon rank sum test; Pearson’s Chi-squared test; Fisher’s exact test. ^3^ The presence of necrotizing enterocolitis refers to necrotizing enterocolitis (>Bells Stage II) at any time point prior to definitive closure.

**Table 4:** Sub-group analysis from Table 3 to match the procedure body weight; only procedure weight under 1200 g.

Characteristics	Device ^1^ (N = 40)	Ligation ^1^ (N = 19)	*p*-Value ^2^
Demographics			
Gestational age (weeks)	24.50 (24.00, 25.00)	24.00 (24.00, 25.00)	0.680
Birth weight (grams)	695 (630, 813)	680 (645, 765)	0.903
Birth weight Z-scores	0.34 (−0.37, 1.09)	0.33 (−0.09, 0.78)	0.772
Sex (female)	28 (70%)	9 (47%)	0.093
Age at procedure (days)	26 (21, 36)	24 (18, 31)	0.149
Postmenstrual age at procedure (weeks)	29.5 (28.3, 31.0)	28.5 (27.8, 30.0)	0.107
Weight at procedure (grams)	1000 (900, 1100)	890 (723, 1000)	0.008
Procedure weight Z-scores	−0.86 (−1.11, −0.56)	−1.05 (−1.41, −0.72)	0.149
Pharmacotherapy before closure	36 (90%)	15 (79%)	0.416
#of pharmacotherapy courses	2.00 (1.00, 3.00)	2.00 (1.00, 2.00)	0.442
Common comorbidities prior to closure			
Necrotizing enterocolitis (≥Bells Stage II)	4 (13%)	6 (32%)	0.151
Intraventricular hemorrhage (any IVH) Grade III/IV IVH	18 (47%)	10 (52%)	0.708
Baseline respiratory/hemodynamic status			
Oxygen saturation index	4.5 (3.8, 6.3)	5.0 (2.0, 6.8)	0.872
Use of inotropic	3 (16%)	2 (18%)	1.00
Post-procedural respiratory outcomes			
High frequency ventilation	8 (25%)	8 (44%)	0.157
Duration of mechanical ventilation (d)	24 (14, 28)	16 (7, 29)	0.647

^1^ Data is reported as. Median (IQR) or n (%). ^2^ Wilcoxon rank sum test; Pearson’s Chi-squared test; Fisher’s exact test.

## Data Availability

The data presented in this study are available on request from the corresponding author.

## Supplementary Materials

The following supporting information can be downloaded at: https://www.mdpi.com/article/10.3390/jcdd11050132/s1, Supplementary Table S1: Demographic and clinical comparisons by indication for surgical ligation.
