# Supplementary material for: Who Still Gets Ligated? Reasons for Persistence of Surgical Ligation of the Patent Ductus Arteriosus Following Availability of Transcatheter Device Occlusion for Premature Neonates
Source: J Cardiovasc Dev Dis. 2024 Apr 23;11(5):132. doi: 10.3390/jcdd11050132 (PMC11122133; doi:10.3390/jcdd11050132)
Supplement: Supplementary file 1 [file jcdd-11-00132-s001.zip › jcdd-2935431-supplementary.pdf]

Characteristics	Abdominal pathology [1] N=7	Unfavorable PDA anatomy[2] N=8	Active infection[3] N=9	p-value
**Demographics**				
Gestational age (weeks)	25 (24, 26)	25 (24, 28)	24 (23, 24)	0.112
Birth weight (grams)	680 (620, 793)	710 (600, 951)	670 (640, 690)	0.751
Birth weight Z-scores	-0.01 (-0.14, 0.23)	-0.48 (-0.94, 0.78)	0.44 (0.18, 0.74)	0.418
Sex (female)	3 (43%)	6 (75%)	3 (33%)	0.266
Age at procedure (days)	19 (14, 21)	41 (34, 75)	39 (25, 43	0.010
Postmenstrual age at procedure (weeks)	28 (28, 31)	27 (0, 32)	28 (27, 29)	0.687
Weight at procedure (grams)	745 (665, 875)	1,550 (975, 2,150)	910 (800, 1,185)	0.021
Procedure weight Z-scores	-1.38 (-1.43, -0.99)	-1.14 (-2.72, -0.78)	-0.98 (-1.49, -0.69)	0.915
Pharmacotherapy before closure	4 (57%)	7 (88%)	7 (78%)	0.432
#of pharmacotherapy courses	1 (0, 2)	2 (1, 2)	2 (1, 2)	0.475
**Common comorbidities prior to closure**				
Necrotizing enterocolitis (≥ Bells Stage II)	6 (86%)	0 (0%)	0 (0%	0.000
Intraventricular hemorrhage (any IVH)<br>Grade III/IV IVH	3 (43%)<br>2 (29%)	1 (14%)<br>0 (0%)	6 (67%)<br>3 (33%)	0.126<br>0.314
**Post-procedural respiratory outcomes**				
High frequency ventilation	3 (43%)	2 (29%)	2 (25%)	0.855
Duration of mechanical ventilation (days)	8 (5, 15)	26 (13, 30)	43 (14, 70)	0.327

Data is reported as n (%)

PDA, patent ductus arteriosus

1Defined as Bells Stage II necrotizing enterocolitis within two weeks of referral or spontaneous intestinal perforation with a Penrose drain

2Defined as echocardiographic evidence of cortication of the aorta or left pulmonary artery stenosis, and a ductus that was < 3 mm in length and < 4 mm in diameter

3Defined as culture positive bacteremia and on antibiotics at the time of definitive closure consideration

Online Supplement Table S1: Demographic and clinical comparisons by indication for surgical ligation
